# Complete Genome Sequence of a Ranavirus Isolated from Chinese Giant Salamander (*Andrias davidianus*)

**DOI:** 10.1128/genomeA.01032-13

**Published:** 2014-01-09

**Authors:** Na Wang, Min Zhang, Lifeng Zhang, Hongli Jing, Yulin Jiang, Shaoqiang Wu, Xiangmei Lin

**Affiliations:** Institute of Animal Quarantine, Chinese Academy of Inspection and Quarantine, Beijing, Chinaa; Beijing Entry-Exit Inspection and Quarantine Bureau Technology Centre, Beijing, Chinab

## Abstract

A ranavirus (RV) was isolated from Chinese giant salamanders (*Andrias davidianus*) in China in 2010 and provisionally designated *Andrias davidianus* ranavirus (ADRV). The complete genome sequence is 106,719 nucleotides long. Genomic sequence and phylogenetic analyses showed that ADRV has a high degree of conservation with other RVs.

## GENOME ANNOUNCEMENT

*Ranavirus*, one of the genera of the *Iridoviridae* family, is recognized as causing an emerging infectious disease that is included in the list of notifiable diseases by the World Organisation for Animal Health (OIE). Ranavirus (RV) infections have been associated with mass mortalities of several amphibian, reptile, and fish species worldwide (1). In July 2010, an RV was isolated from diseased Chinese giant salamander (*Andrias davidianus*), which is one of the world’s largest amphibian species and is classified as critically endangered by the International Union for the Conservation of Nature and Natural Resources; this virus was provisionally designated *Andrias davidianus* ranavirus (ADRV) (2).

To obtain the complete genome sequence of ADRV, the virus was cultured on bluegill fry (BF2) cells, and DNA from purified viral particles was extracted as described previously (3). Sequencing was performed using a 454 GS FLX Titanium sequencing system (Roche) combined with Sanger sequencing of the PCR products for unresolved regions. Newbler Assembler software (454 Life Sciences) was used to assemble the data into unordered and unoriented contigs. Open reading frames (ORFs) were predicted with Glimmer 3.0 and Prodigal version 1.2 (http://compbio.ornl.gov/prodigal/server.html). Annotation of the predicted ORFs was conducted using BLASTp searches (http://blast.ncbi.nlm.nih.gov/Blast.cgi) and RPS-BLAST searches (http://www.ncbi.nlm.nih.gov/Structure/cdd/wrpsb.cgi) at the NCBI Conserved Domain Database (CDD) and Clusters of Orthologous Groups of proteins (COG) database.

The ADRV genome has a full length of 106,719 bp, with a G+C content of 55.04% and 101 ORFs encoding putatively expressed proteins with conserved domains assigned to structural proteins, as well as proteins potentially involved in replication, transcription, and host response modification, which possess defined functions and are highly homologous to those of other RVs. For 95 ADRV-predicted proteins, orthologs were readily found in other RV genomes, with identities ranging from 77% to 100%. Only six ORFs had not been previously annotated in other RVs, but no significant similarities to other known proteins were found in the databases. Whether these proteins are truly expressed and functional during ADRV infection remains to be addressed.

The phylogenetic relationships between ADRV and other fully sequenced RVs show that ADRV has a high degree of similarity to other RVs, which are closely related to the type virus of the genus *Ranavirus*, frog virus 3 (FV3) (4). The degree of colinearity analysis performed by using dot plot analyses of the ADRV genome with those of other RVs revealed similar results (data not shown). Interestingly, the ADRV genome shows complete colinearity with the genome of common midwife toad virus (CMTV), isolated from diseased tadpoles in 2007 in Spain (GenBank accession no. JQ231222) (5). Studies investigating the complete genome of ADRV will provide novel information about RVs and are beneficial for investigating the epidemiology and ecology of RVs worldwide.

### Nucleotide sequence accession number.

The complete genome sequence of ADRV has been deposited in GenBank under the accession no. KF033124.
